# Missing Eutectic Transition in Electrolyte Solutions in Confinement Due to Ion Accumulation in the Interfacial Layer

**DOI:** 10.1021/acs.jpcb.5c01621

**Published:** 2025-07-15

**Authors:** Shaoheng Wang, Michael Steiger

**Affiliations:** Department of Chemistry, 14915 University of Hamburg, Martin-Luther-King-Platz 6, Hamburg 20146, Germany

## Abstract

The anomalous properties of aqueous electrolytes under nanoconfinement are of wide concern due to their widespread occurrence in both natural and industrial processes. Recent studies suggest that the eutectic transition of dilute solutions in nanopores is inhibited. In this work, we propose that the preferential accumulation of dissolved ions in the interfacial layer and the resulting depletion of the ions in the core of the pore solution are the key factors contributing to the absence of salt crystallization at the eutectic point. Systematic calorimetric measurements were conducted with CaCl_2_ and NaCl solutions confined in nanoporous silica. The results demonstrate that the occurrence of the eutectic transition is controlled by three main factors: salt concentration, pore size, and pore filling degree. In confined solutions with an insufficient supply of ions, the interfacial layer remains unsaturated, thus leading to the crystallization of solely ice in the core and the absence of a eutectic transition. Conversely, in more concentrated solutions or if the supply of ions is sufficient, the interfacial layer becomes saturated, and the presence of excess bulk-like ions in the core facilitates simultaneous salt and ice crystallization at the eutectic point. Supply for the complete saturation of the interfacial layer can stem either from a sufficiently large core volume, thus, in large pores, or from diffusion from a reservoir outside of an overfilled pore. These findings highlight the critical role of interfacial layers influencing the ion distribution and phase behavior of electrolyte solutions in nanoconfinement, specifically confirming sorption and strong enrichment of ions in the interfacial layer.

## Graphical abstract

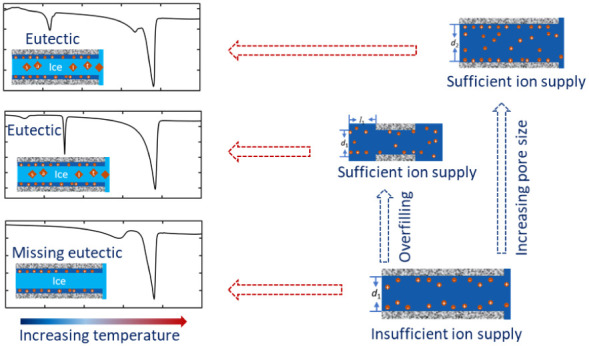

## 1 Introduction

The impact of confinement on the phase transition of water or electrolyte solutions has attracted significant attention due to their frequent occurrence in natural processes and technological applications.^1–4^ Specifically, phenomena such as the freezing and melting of water and vapor condensation have been extensively studied.^5–8^ A widely accepted perspective suggests that the freezing and melting points of water decrease as pore size decreases, and the first-order phase transition is suppressed when pore diameters fall below 2.5 nm.^8^ Recently, increasing interest has also been directed toward the phase transition of aqueous solutions,^9–14^ as these scenarios better represent real-world conditions compared to pure water. Freezing or salt crystallization from solution is a continuous process along the solid–liquid phase boundary over a specific temperature range. This process begins with either ice or salt crystallization and ends with the simultaneous crystallization of both solids at the eutectic point, which marks the final state of phase equilibrium before complete solidification.^9^ The eutectic point represents the lowest temperature at which a liquid solution remains thermodynamically stable and marks the beginning of melting in a salt–ice mixture. In confinement, the eutectic point in electrolyte solutions is significantly shifted to lower temperatures.^9,10,12^ Consequently, the eutectic melting of confined brines is considered the most plausible mechanism for the presence of liquid water on Mars. Theoretically, the eutectic transition should occur in all salt solutions, regardless of the solute type or concentration. However, under confinement, this transition was only observed in concentrated solutions but was absent in dilute solutions in previous studies.^15,16^ To date, this anomalous behavior lacks a clear and comprehensive explanation. This phenomenon is apparently similar to the nonfreezing behavior observed in pure water within pores smaller than 2.5 nm or under partial filling conditions, attributed to the strong interaction of water molecules with the pore wall.^5,8,17–20^ Meissner et al.^9^ proposed that ions crystallize as extremely small nanocrystals within the interfacial layer, producing a secondary confinement effect on the remaining solution. Argyris et al.,^21^ using molecular dynamics simulations, found strong accumulation of dissolved ions in the interfacial layer of silica nanopores. In this perspective, ions can be categorized based on their spatial distribution: interfacial ions located near the pore wall and bulk-like ions in the core of the pore.

It is well-established that water molecules are unable to freeze within an interfacial layer of about 0.6 nm thickness.^8^ Ion crystallization in this region is likely inhibited as well. Consequently, the eutectic transition can occur only in the core of the pore solution and only if the accumulation of ions in the interfacial layer leaves a sufficient amount of ions in the core. The total number of ions is influenced by three factors: salt concentration, pore volume, and pore filling degree. These factors directly determine the existence of bulk-like ions in the core, which results in the eutectic transition. In this study, systematic calorimetric measurements were conducted to investigate the effects of salt concentration, pore size, and pore filling degree on the eutectic transition in nanoporous silica. The results confirm the strong adsorption and accumulation of ions within the nonfreezing interfacial layer, meaning that no bulk-like ions are present in the core, thus impeding the eutectic transition. Only if the interfacial layer is saturated can the eutectic transition in dilute solutions be observed.

## 2 Materials and Methods

### 2.1 Mesoporous Materials

Porous SBA-15 silica was prepared as described by Zhao et al.^22^ In a typical synthesis, the triblock copolymer Pluronic p-123 (*M*_av_ = 5800, EO_20_PO_70_EO_20_, Aldrich) was used as a structure-directing agent, with tetraethoxysilane (TEOS, Aldrich, 98%) serving as the silica precursor. Further details are provided in a previous study by Talreja-Muthreja et al.^14^ The siliceous mesostructured cellular foam (MCF) used in this work was the same as used in the previous study.^14^ The porous silica samples were characterized using nitrogen adsorption, and pore diameters, specific surface areas, and specific volumes were calculated using the NLDFT method for cylindrical pores. The results are presented in Figure 1 and Table 1.

**Figure 1:**
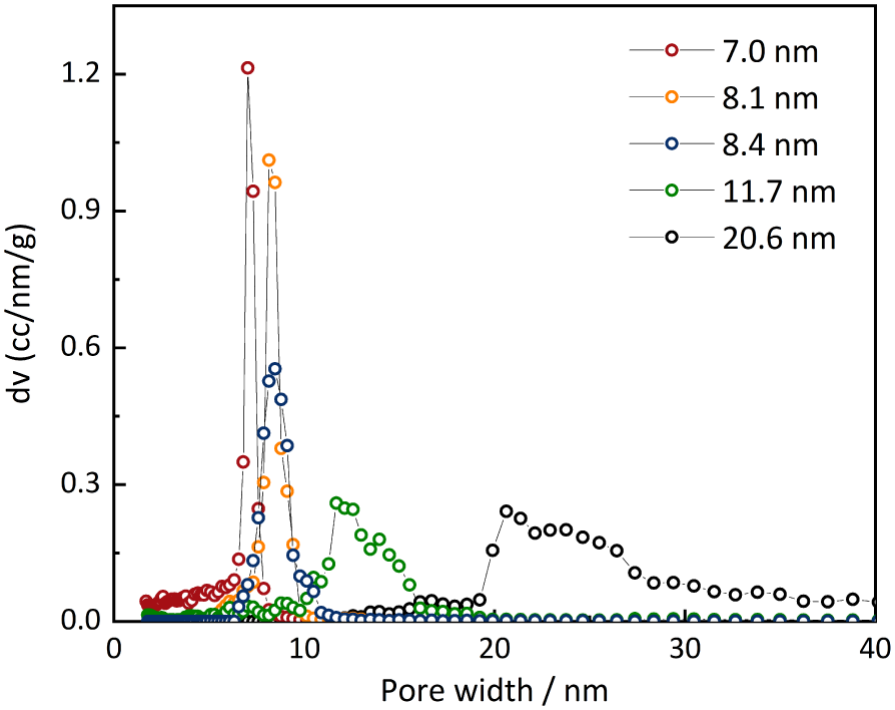
Pore size distributions of the SBA-15 silica (7.0–11.7 nm) and the MCF silica (20.6 nm).

**Table 1:** Pore Diameters *d*, Specific Surface Areas *S,* and Specific Volumes *V* of the Porous Silica Materials^a^

Material	*d*/nm	*v*/cm^3^·g^–1^	*s*/m^2^·g^–1^
SBA-15	7.0	1.12	834
SBA-15	8.1	1.15	570
SBA-15	8.5	1.05	484
SBA-15	11.7	1.25	452
MCF	20.6	2.71	423

a MCF sample from reference 14.

### 2.2 Calorimetric Measurements

The samples were prepared by impregnation of a weighed mass of the silica material (approximately 50 mg) with an appropriate amount of solution (CaCl_2_ or NaCl, >99%, Merck, Germany) in a mortar. Grinding facilitated the formation of a homogeneous mixture of the liquid and the porous material. The filling degree was estimated by the ratio of the solution volume to the pore volume of the respective material. Calorimetric measurements were conducted using a Setaram BT 2.15 calorimeter in DSC mode with very low cooling and heating rates of 0.1 K·min^–1^ with a 3 h hold time at the lowest temperature. The very low heating rates were selected to minimize kinetic influences in the melting scans. The working temperature range depends on the intrinsic properties of salt and pore size, spanning from −100 to 25 °C for CaCl_2_ solutions and from −80 to 25 °C for NaCl solutions.

## 3 Results and Discussion

### 3.1 Eutectic Transition in Bulk and in Confined Solutions

The freezing or melting of a salt solution is more complex than that of pure water, as it involves a phase separation of both ice and salt crystals from the solution. As shown in Figure 2a, for a solution with a concentration lower than the eutectic concentration (solution A), the freezing process begins with the crystallization of ice at the freezing temperature (point B). Upon further cooling, ice crystallization continues, and the concentration in the remaining solution increases until the eutectic point E is reached. The solution is now also saturated with the salt, and further cooling causes the simultaneous crystallization of both solids. The reverse process starts with eutectic melting. In the case of the frozen solution A, the salt dissolves completely in the eutectic melt, and upon further heating, ice melting continues until point B is reached.

**Figure 2:**
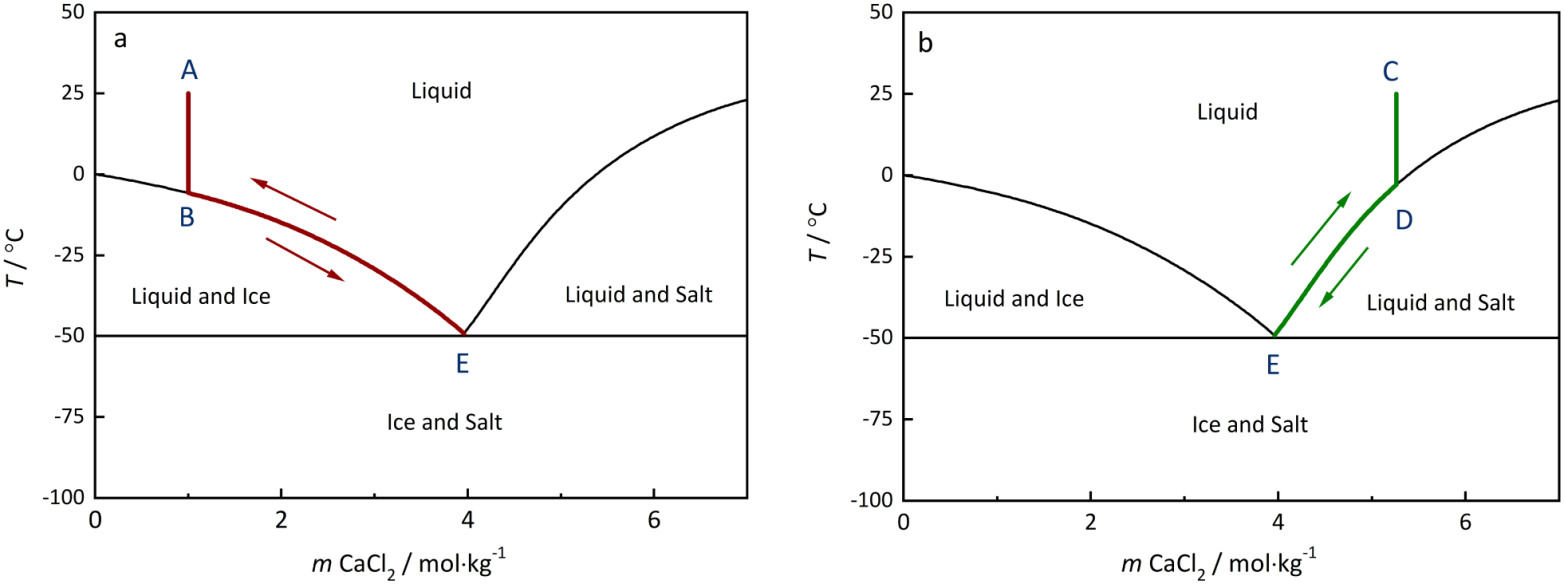
Equilibrium freezing and melting pathways of bulk CaCl_2_ solutions. (a) Starting from solution A with a concentration lower than the eutectic concentration. (b) Starting from solution C (concentration higher than the eutectic concentration). Solid lines are freezing temperatures and solubilities of CaCl_2_·6H_2_O calculated with the model of Wang et al.^23^ which is based on critically evaluated freezing temperatures and solubilities.

The freezing and melting process of a solution with a concentration higher than the eutectic concentration (solution C) is illustrated in Figure 2b. In this case, the process starts with crystallization of the crystalline salt (point D). Upon further cooling, the salt crystallizes continuously until the eutectic is reached, below which both solids crystallize (point E). The melting process of this mixture starts with complete ice melting at the eutectic temperature and partial dissolution of the salt. During subsequent heating, the dissolution of the salt continues until point D is reached. A similar diagram for the phase transition pathways of NaCl solutions is shown in Figure S1 (Supporting Information).

A typical thermogram of a DSC heating scan of a frozen bulk 0.4 m CaCl_2_ solution yields two endothermic signals (Peaks 1 and 2), as illustrated in Figure 3a. Peak 1 corresponds to the eutectic melting of the solid mixture of CaCl_2_·6H_2_O and ice at the eutectic temperature of −50 °C (corresponding to point E in Figure 2a). Peak 2 represents the temperature at which ice melting is complete (corresponding to point B in Figure 2a). This latter transition is termed the ice melting temperature in the following discussion to distinguish it clearly from the eutectic point. It should be noted, however, that the eutectic melting Peak 1 represents a transition at a single temperature, thus yielding a sharp peak. The peak width in a bulk solution is controlled essentially by the properties of the calorimeter and the heating rate. In confinement, the shape of the eutectic melting peak is controlled by the pore size distribution.^8^ In contrast, the ice melting Peak 2 represents the continuous process of ice melting along the line EB in Figure 2a, starting right above the eutectic temperature and extending to the end point B, the temperature of which depends on the solution composition.

**Figure 3:**
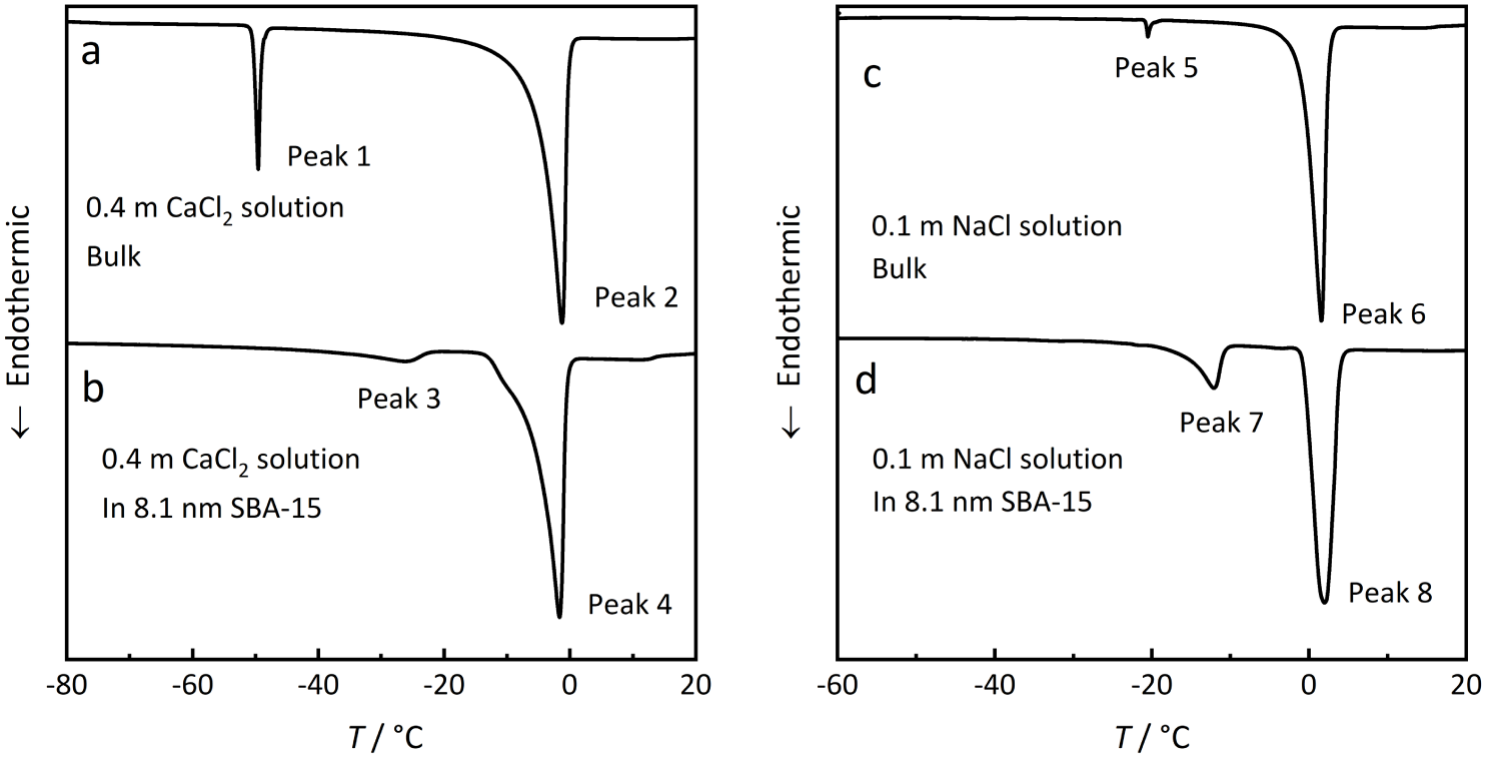
Melting curves of dilute 0.4 m CaCl_2_ (a,b) and 0.1 m NaCl (c,d) solutions both in bulk and in 8.1 nm SBA-15 confinement (overfilling 150%); the total amount of solution was the same in the bulk measurement and in the experiment in confinement.

DSC scanning of the same amount of the 0.4 m CaCl_2_ solution in an 8.1 nm SBA-15 sample with 150% filling degree is shown in Figure 3b. The overfilled confined solution also exhibits two thermal signals (Peak 3 and Peak 4) during the melting process. The higher-temperature signal, Peak 4, closely aligns with Peak 2 of Figure 3a, representing the melting of bulk ice in the salt solution. However, compared to Peak 1 (bulk eutectic point), Peak 3 is shifted significantly to a higher temperature. This suggests that Peak 3 reflects the ice melting within the pore rather than the eutectic transitions, which are expected at −50 °C for the bulk solution (Peak 1) and at lower temperature in the pore as discussed below.

Similar results are observed in measurements with a dilute 0.1 m NaCl solution in bulk and in confinement (Figure 3c,d). Peaks 5 and 6 represent the eutectic transition and the continuous ice melting, respectively, in the bulk solution, while the Peaks 7 and 8 correspond to ice melting occurring in the bulk and within the pore, respectively. Thus, in both experiments with rather dilute solutions, no signal of a eutectic transition is observed. Since the total amounts of the CaCl_2_ and NaCl solutions were the same in the bulk and in confinement, these findings clearly confirm that the absence of a detectable eutectic transition in confined dilute solutions is a real effect rather than an experimental artifact due to the limited sensitivity of the calorimetric measurement as suggested in a previous investigation.^16^

### 3.2 Influence of Salt Concentration

The absence of the eutectic melting signal is observed only with dilute electrolyte solutions. In contrast, it is well-known that more concentrated solutions yield eutectic melting signals in pores that are shifted to lower temperature than the respective bulk eutectics.^9,10,12,15^ Figure 4 presents a systematic investigation of the influence of solution concentration on the melting curves of frozen solutions of CaCl_2_ (Figure 4a) and NaCl (Figure 4b) in SBA-15 with pore sizes of 9.4 and 8.5 nm, respectively. To ensure complete pore impregnation, all host materials were treated with a solution volume of 150% of the pore volume, resulting in composites that include both bulk and confined solutions. Consequently, the melting curves incorporate phase transition signals from the bulk phases and the confined solutions.

**Figure 4:**
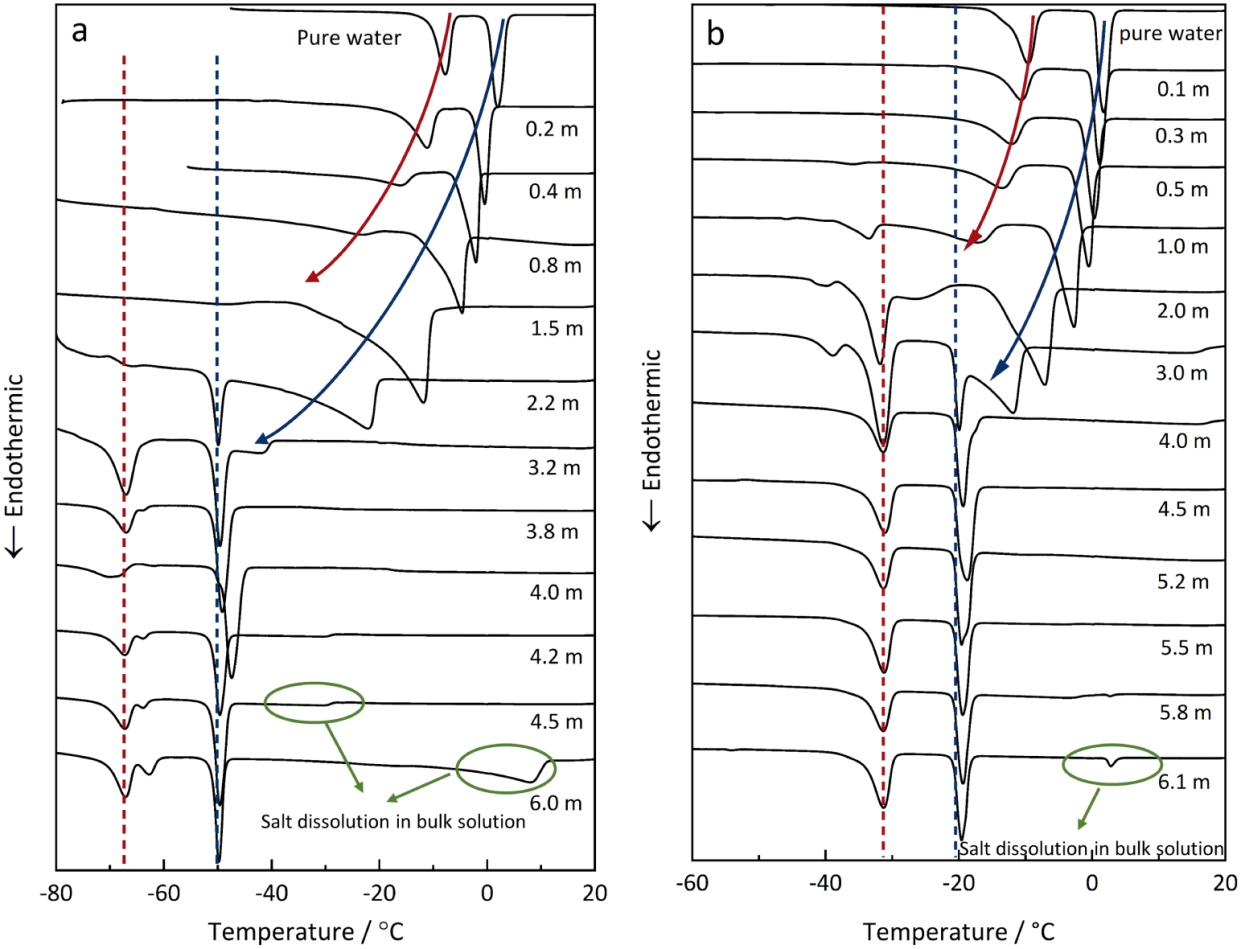
Melting curves of CaCl_2_ solutions (a) in 9.4 nm SBA-15 with molalities 0–6.0 m and NaCl solutions (b) in 8.5 nm SBA-15 silica with molalities 0–6.1 m (overfilling 150%). The dashed red and blue lines indicate in-pore and bulk eutectic points, respectively; red and blue arrows are drawn to guide the eyes along the end points of the ice melting in pore and in bulk, respectively.

The influence of salt concentration on the melting process of the frozen CaCl_2_ and NaCl solutions follows a similar pattern with distinct behavior observed in both the low- and high-concentration regions. For CaCl_2_, the melting curves for concentrations at 1.5 m and below show only two endothermic peaks, corresponding to the ice melting in the bulk phase and in the confined solution, as discussed in the previous section. The temperature of both peaks decreases with increasing concentration, as illustrated by the blue (bulk) and the red (confinement) arrows in Figure 4a. The temperature decrease illustrated by the arrows reflects the influence of concentration on the ice melting temperature, as shown in Figure 2a. The shift between the ice melting temperatures in pore (red arrow) and in bulk (blue arrow) reflects the decrease in the melting temperatures due to the limited size of the ice crystals in confinement. Notably, no eutectic transition is detected in either bulk phase or in the confined solution at these concentrations.

At high concentrations (2.2 m and above), two endothermic peaks at approximately −67 °C (dashed red line) and −50 °C (dashed blue line) indicate eutectic melting in confinement and in the bulk, respectively. It should be noted that the bulk eutectic temperature is represented by the peak onset temperature in DSC, while the eutectic temperature in confinement is better represented by the peak maximum.^8^ A significant depression of the eutectic melting temperature due to the confinement effect is evident, independent of the salt concentration. The smaller peaks (illustrated by the green circles in Figure 4a) above the bulk eutectic temperature correspond to continuous salt dissolution, which follows the pathway illustrated in Figure 2b (along line ED) at concentrations above the eutectic concentration (e.g., 4.5 and 6.0*m* in Figure 4a).

It is notable that the ice melting peak of the confined solutions does not appear in the thermograms. This absence is attributed to ion diffusion and exchange between solutions inside and outside of the pore during the freezing process, thus leading to salt fractionation and a final ice–salt mixture composition in the pores that matches the eutectic composition.^9^ If crystallization in the bulk solution starts with the precipitation of ice (i.e., at concentrations below the eutectic as in Figure 2a), there is a rapid increase in concentration, resulting in inward diffusion. If, on the other hand, crystallization starts with the precipitation of CaCl_2_·6H_2_O, the concentration decreases, resulting in outward diffusion. In either case, the concentration within the pore approaches the eutectic concentration. As a result, thermal signals of ice melting or salt dissolution within the confined solutions do not appear, as illustrated in Figure S2 (Supporting Information). Overall, the salt concentration, which primarily controls the total number of ions in the pore, has a significant impact on the appearance of the eutectic transitions.

Similarly, eutectic transitions are observed only in concentrated NaCl solutions and are absent in dilute solutions, as shown in Figure 4b. The molality threshold for the absence of the eutectic transition in confined NaCl solutions (≤0.5 m) is notably lower than in the case of CaCl_2_ (≤2.2 m), indicating a stronger influence of confinement on the phase behavior of the CaCl_2_ solution. This disparity is likely due to the stronger interaction between the ions and the pore wall. It is well established that the highly polarized silica-solution interface is negatively charged, primarily resulting from the deprotonation of the silanol group upon contact with solutions.^24,25^ The deprotonation equilibrium and surface charge density are affected by both pH and salt concentration. According to the classic Poisson–Boltzmann (PB) theory, this results in the formation of an electrical double layer (EDL), characterized by counterion accumulation and co-ion depletion.^26^ Ion enrichment near the interface has been well investigated by Monte Carlo and molecular dynamics simulations, showing significant ion accumulation within a layer of approximately 1 nm from the pore wall.^21,27–30^

Based on these insights, we propose that ion accumulation in the interfacial layer at the pore walls, where the first-order phase transition disappears, leads to significant depletion of ions in the core solution, resulting in the absence of the eutectic transition. The ion enrichment behavior is likely sensitive to the ionic charge, as the electrostatic attraction between the negatively charged pore wall and a divalent cation (Ca^2+^) is stronger than with a monovalent cation (Na^+^). This stronger interaction leads to a higher capacity for divalent ions to accumulate in the interfacial layer, thereby explaining why the eutectic transition in confined CaCl_2_ solutions occurs only at higher concentrations compared to NaCl.

In the experiments presented here with dilute solutions, the limited number of ions is insufficient to saturate the interfacial layer. Thus, all available ions are consumed completely, leading to the absence of salt crystallization and eutectic transition. In contrast, in the more concentrated solutions, there is a sufficient supply of ions to saturate the interfacial layer, and the excess bulk-like ions in the core solution crystallize at the eutectic point.

### 3.3 Pore Filling Degree and Pore Size

The key factor influencing the phase behavior of the confined solution is thus the total amount of ions present rather than the apparent salt concentration. In addition to the initial concentration, the total reservoir of dissolved ions is also controlled by both the pore size and the degree of pore filling. Figure 5 illustrates the heat flow curves during the melting of frozen CaCl_2_ and NaCl solutions in SBA-15 and MCF silica with different pore sizes and overfilling degrees. Red and black curves represent DSC scans with and without detection of the eutectic transition, respectively. As shown in Figure 5a for 8.5 nm pores impregnated with a 0.8 m CaCl_2_ solution at pore filling degrees of 150%, 450%, and 750%, the in-pore eutectic transition is observed only in the 750% overfilled sample but not in the other two samples. A similar effect of pore filling degree on the eutectic transition is also obvious in the NaCl samples, as shown in Figure 5e. The bulk solution connected to the pores serves as a reservoir ensuring supply with a sufficient amount of ions to fill the interfacial layer while still maintaining a sufficiently high concentration in the core to observe the eutectic transition. A few experiments were carried out with dilute NaCl solutions and pore fillings below 100%. The resulting DSC scans revealed only a single broad ice melting peak and, as expected, no signal for the eutectic transition. Therefore, further experiments with low pore fillings were not conducted.

**Figure 5:**
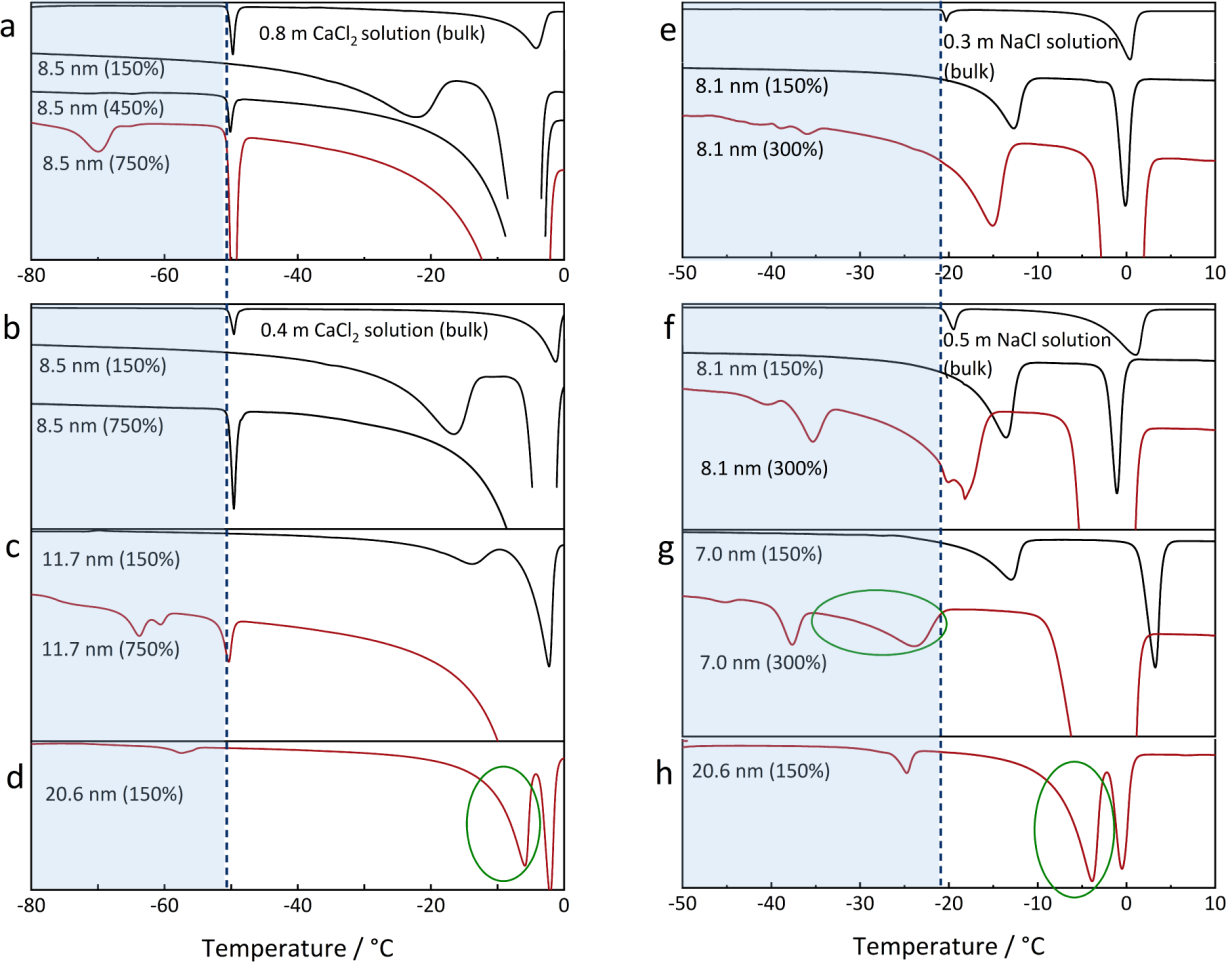
Heat flow curves during melting of CaCl_2_–H_2_O (a–d) and NaCl–H_2_O (e–h) in SBA-15 silica (7.0–11.6 nm) and a siliceous mesostructured cellular foam (MCF, 20.6 nm) with different pore sizes and pore filling degrees (150%–750%). Vertical dashed lines: the bulk eutectic temperatures; blue areas: the regions where the signal of the eutectic transition in the confined solution is expected; red and black curves: measured thermograms with and without detection of the eutectic transition.

Finally, alongside the salt concentration and pore filling degree, pore size is the third factor affecting the total amount of salt in the confined solution. Figure 5b–d compares the melting curves for different pore sizes at a constant pore filling degree. At 150% pore filling, the eutectic transition occurs only in the 20.6 nm pore and is absent in the smaller pores (11.7 and 8.5 nm). At 750% pore filling, the eutectic transition is observed in the 11.7 nm pore but not in the 8.5 nm pore. Similar behavior is shown for NaCl solutions in Figure 5f–h. Notably, in some cases where a peak for the eutectic transition in the pore is observed (indicated by the red curves), the expected thermal signal at bulk eutectic temperature did not appear. Instead, signals (marked with green circles) are detected at temperatures deviating from the bulk eutectic point, as seen in Figure 5d,g, and h. As illustrated in Figure 2, ice melting in the salt solution is a continuous process over a temperature range, typically producing a broad thermal peak. In contrast, the eutectic transition takes place at a fixed temperature and is characterized by a sharp, distinct peak. Therefore, these unidentified signals are assigned to ice melting in the confined salt solution, as their broad profiles, as discussed before, are characteristic of gradual thermal events, such as ice melting above the eutectic in the electrolyte solutions. This indicates that the final ice–salt mixture composition within the pore has a lower salt content than the bulk eutectic mixture, even if all ions diffuse into the pore from the bulk solution.

### 3.4 Ion Accumulation in the Pore

To further analyze the ion enrichment in the interfacial layer semiquantitatively, the concentration of CaCl_2_ in this region was calculated based on the following assumptions: (1) the thickness of the interfacial layer is 0.6 nm, consistent with that of pure water, (2) the density in this region is the same as in the core solution, (3) all ions accumulate in the interfacial layer, leaving the pore core devoid of ions, and (4) the total capacity of this layer is limited, and the average concentration does not exceed the eutectic molality of 4*m*. Details of the calculation are provided in Section S3 of the . The results, shown in Figure 6a, display the volume fractions of the interfacial layer (black curve) and the core (gray curve) as functions of the pore diameter. The decrease in the volume fraction of the core solution with decreasing pore size results in a diminished influence of bulk-like ions in the core on the overall properties of the system, making the interfacial ions predominant for pore sizes below 4 nm (the intersection of black and gray curves). In such small pores and at low to moderate concentrations, the interfacial layer remains unsaturated even after collecting all ions from the core solution. The dashed lines represent the interfacial layer concentration as a function of pore size at specific initial solution molalities and 100% pore filling. The intersections of the dashed lines with the blue area boundary—thus, the maximum concentration in the interfacial layer, marked by red, blue, and green circles—yield the threshold pore sizes at which the interfacial layer just reaches saturation at the given initial molalities. At this moment, all ions are consumed to fill up the interfacial layer, and the core consists of pure water. For a 0.4 m CaCl_2_ solution, the threshold pore size is 23.5 nm, in reasonable agreement with our measurements for the 20.6 nm pore size.

**Figure 6:**
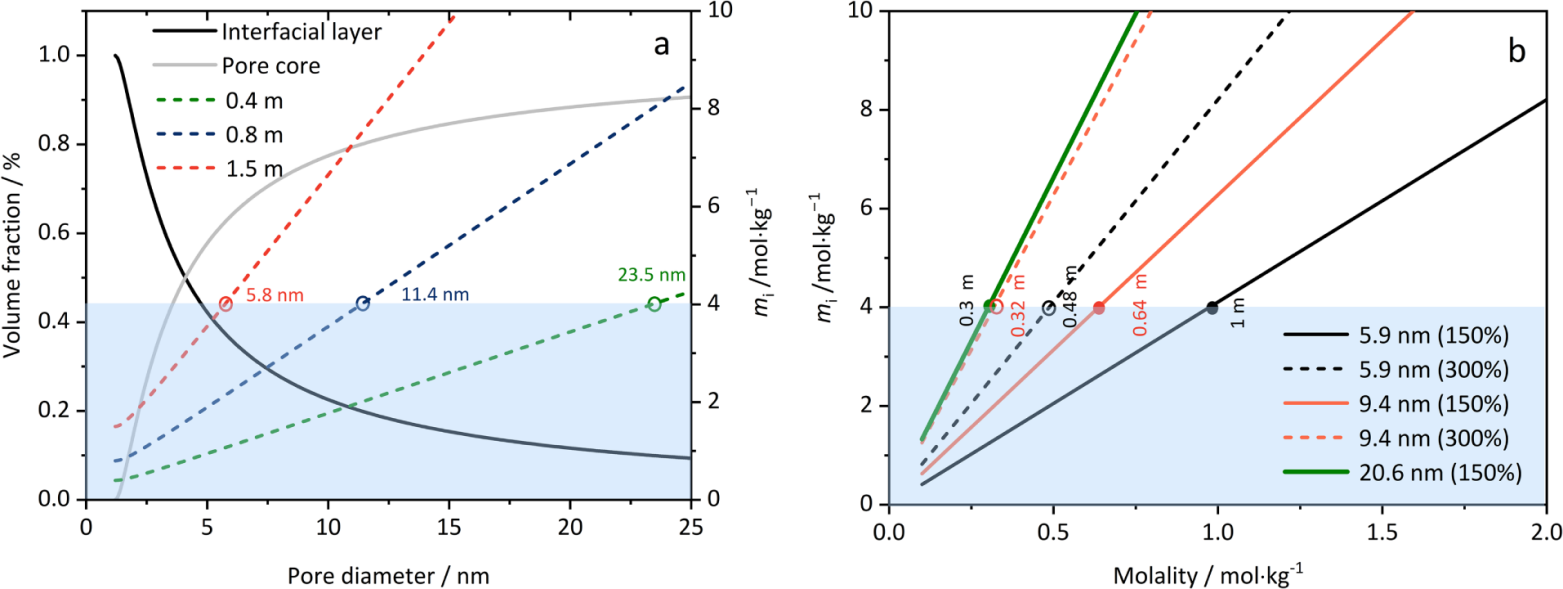
(a) Volume fractions of the interfacial layer (solid black curve) and of the core (solid gray curve), molalities in the interfacial layer (*m*_i_) for initial molalities of 0.4 m (dashed green curve), 0.8 m (dashed blue curve), and 1.5 m (dashed orange curve). (b) Salt concentrations in the interfacial layer for pore sizes of 5.9 nm (solid black line: 150% pore filling; dashed black line: 300% pore filling), 9.4 nm (solid orange line: 150% pore filling; dashed orange line: 300% pore filling), and 20.6 nm (solid green line: 150% pore filling). Blue area: unsaturated interfacial layer region (molality in the interfacial layer below the eutectic molality).

Figure 6b illustrates the relationship between the concentration in the interfacial layer and the initial salt molality for fixed pore sizes and pore fillings. The minimum concentrations at which the interfacial layer reaches saturation at these pore sizes and pore fillings are marked by the green, orange, and black symbols at the upper boundary of the blue area. Only if the initial concentration exceeds these values do dissolved ions remain in the core solution. For example, the limiting concentrations are 0.3 and 0.64 m for 20.6 and 9.4 nm pores at 150% filling, respectively. An increase in the pore filling degree promotes the saturation of the interfacial layer even at low concentration, as shown in Figure 5b for two different pore sizes (open and filled symbols in Figure 6b).

The role of the interfacial layer in the freezing process of dilute solutions within nanopores is illustrated schematically in Figure 7. In a confined solution with a limited reservoir of dissolved ions, such as in a small pore, at low concentration, or at low pore overfilling, the ions are exclusively present within the unsaturated interfacial layer, and the core of the pore consists of pure water (or an extremely dilute solution). Thus, only ice crystallizes in the core resulting in the absence of a eutectic transition (Figure 7a). Conversely, in confined solutions with a sufficiently large reservoir of ions, which can be achieved by larger pore sizes, higher concentrations, or larger overfilling, as shown in Figure 7b–d, the interfacial layer becomes fully saturated, and an excess of ions remains in the core solution, resulting in the eutectic transition within the core of a nanopore.

**Figure 7:**
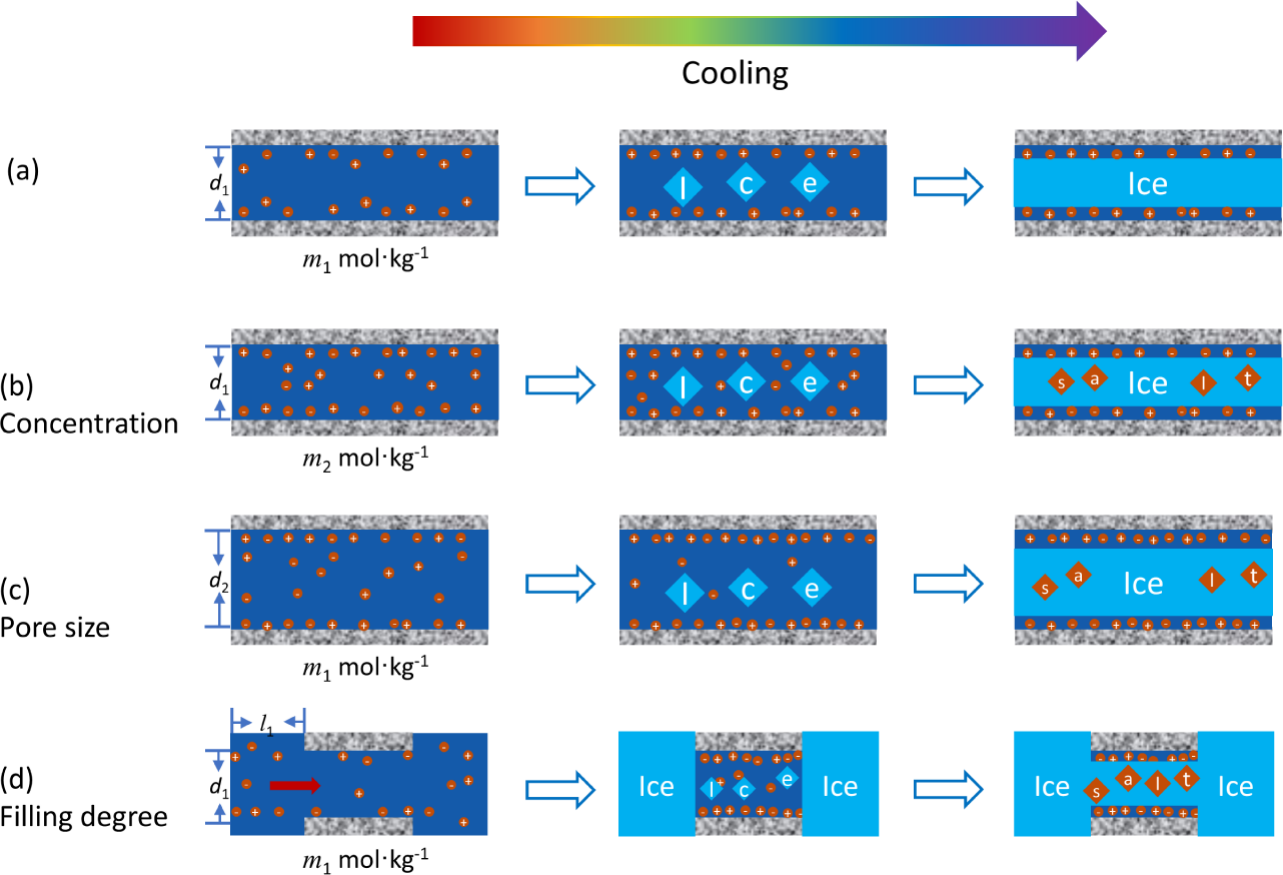
Schematic illustration of freezing of electrolyte solutions in pores. Absence of a eutectic at very low concentrations (a) and formation of a eutectic at larger concentration (b), by increasing the pore size (c) or at larger overfilling (d). Pore sizes: *d*_2_ > *d*_1_; concentrations: *m*_2_ > *m*_1_.

## 4 Conclusions

In summary, anomalous phase behavior of CaCl_2_ and NaCl solutions in nanoconfinement was observed, specifically the absence of a eutectic transition at low concentrations. This study is the first to report how the eutectic transition of confined dilute solutions is controlled by concentration, pore size, and pore filling degree. The findings suggest that strong ordering and preferential enrichment of dissolved ions in the interfacial layer and the resulting depletion of ions in the core solution result in the absence of salt crystallization. Formation of crystalline salt and the eutectic transition only occur when bulk-like ions remain in the core solution, thus, if the interfacial layer is fully saturated. The saturation of the interfacial layer depends on the total ion content, which is determined by three factors: salt concentration, pore size, and pore filling degree. The measurements presented in this work provide solid experimental evidence for the strong accumulation of dissolved ions in the interfacial layer. Furthermore, the temperature depression of the ice melting and eutectic point of electrolytes in nanopores and its pore size dependence are worth further quantitative analysis, as they are significantly affected by the ice–solution interfacial energy.^31^ This discussion will be extended in a subsequent paper that integrates the experimental results with a thermodynamic model. Ion diffusion from the bulk solution into the confined space holds potential for practical applications, such as desalination and enhancing oil recovery.^32,33^ Their findings provide experimental evidence supporting our interpretation of ion accumulation in the interfacial layer and ion exchange between the solution inside and outside the pore.

## Supplementary Material





## Notes

Experimental raw data (XLSX)
Freezing and melting pathways in bulk NaCl solutions and in confined salt solutions with additional figures; details on the calculation of ion accumulation in the interfacial layer (PDF)
