# Supplementary material for: Missing Eutectic Transition in Electrolyte Solutions in Confinement Due to Ion Accumulation in the Interfacial Layer
Source: J Phys Chem B. 2025 Jul 15;129(29):7623–30. doi: 10.1021/acs.jpcb.5c01621 (PMC12302066; doi:10.1021/acs.jpcb.5c01621)
Supplement: Supplementary file 2 [file jp5c01621_si_002.pdf]

Supporting Information

Missing eutectic transition in electrolyte solutions in confinement due to ion accumulation in the interfacial layer

Shaoheng Wang, Michael Steiger*

Department of Chemistry, University of Hamburg, Hamburg, Germany

* Correspondence to: michael.steiger@uni-hamburg.de

1. Freezing and melting pathways in NaCl solutions

Freezing and melting pathways of NaCl solutions are shown in Figure S1. For a solution with a concentration lower than the eutectic composition (solution A), the freezing process starts with the precipitation of ice at the freezing temperature (point B). Upon further cooling, ice crystallization continues and the concentration in the remaining solution increases until the eutectic point E is reached below which a solution is no longer stable and both ice and $NaCl \cdot 2H_2O$ crystallize. The reverse process starts with eutectic melting, i.e. the partial melting of ice and the complete dissolution of $NaCl \cdot 2H_2O$ in the melt. Further heating causes continuous ice melting until point B is reached. For a solution with a concentration higher than the eutectic composition (solution C), the process starts with the crystallization of $NaCl \cdot 2H_2O$ (point D). Further cooling leads to continuous salt crystallization along line DE until the eutectic is reached (point E). In this case, the melting process starts with complete ice melting and partial dissolution of the salt at the eutectic temperature. During subsequent heating, dissolution of the salt continues until point D is reached.

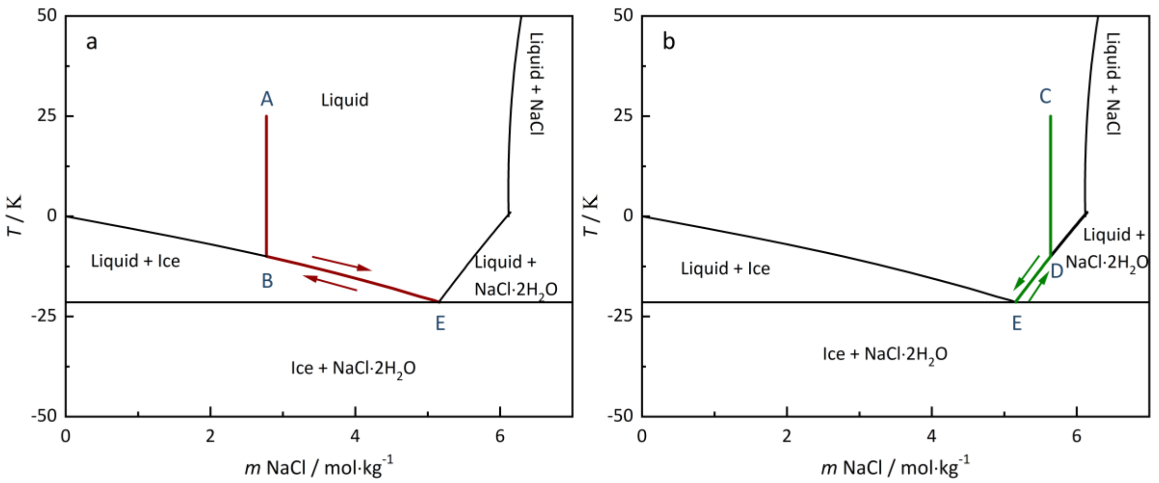

Figure S1. Equilibrium freezing and melting pathways of bulk NaCl solutions. (a) Starting from solution A with a concentration lower than the eutectic concentration. (b) Starting from solution C (concentration higher than eutectic concentration). Solid lines are freezing temperatures and solubilities of $NaCl \cdot 2H_2O$ and NaCl calculated with the same model as used previously,[1] which is based on critically evaluated freezing temperatures and solubilities.

S2. Freezing and melting pathways of a confined salt solution

The freezing and melting process of a confined salt solution in an overfilled pore is illustrated in Figure S2. Colling a solution at composition A, the freezing point of the bulk solution outside the pore is reached at point B. The segment BE represents a continuous freezing process in which ice crystallizes out from the bulk solution. This crystallization leads to an increase of the salt concentration in the remaining liquid. The resulting concentration gradient triggers diffusion of ions which are transported into the pore. Due to ongoing ice precipitation outside and diffusion inwards, the salt concentrations continuously approaches the eutectic composition both in the external solution and within the pore and ice formation occurs exclusively in the bulk during this initial stage.

As temperature and salt molality shifts to point E, the eutectic point of the bulk solution is reached, where both ice and salt begin to crystallize simultaneously in the bulk phase. At this stage, the salt concentration in the confined solution equals the eutectic concentration of the bulk. In confinement, due to the limited crystal sizes, both the freezing curves and the solubility curve of the salt are shifted to lower temperatures as shown by the two dotted lines. There intersection is the eutectic point F within the pore. Thus, upon decreasing the temperature from the bulk eutectic temperature E to the eutectic temperature F within the pore, eutectic crystallization also occurs in the pore, involving the simultaneous formation of ice and salt. Consequently, the salt-to-ice ratio in the pore at this point (i.e., eutectic composition) differs significantly from the initial composition of the solution.

During the reverse melting process, liquefaction begins in the pore at point F, corresponding to the complete eutectic melting of the salt–ice eutectic mixture within the pore, leaving no residual solid in the confined space. As the temperature further increases, eutectic melting in the bulk solution occurs at point E, followed by continuous ice melting along path EB, which is complete at point B.

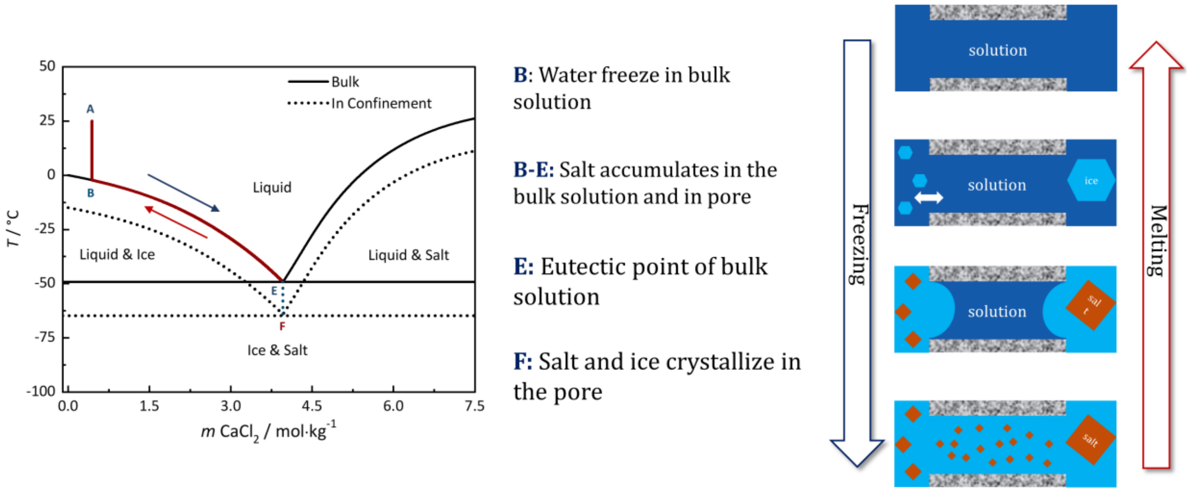

Figure S2. Freezing pathway of the salt solution within the overfilled pores. This diagram indicates that the final ice–salt mixture in the pore is as same as the eutectic concentration, resulting in the absence of ice melting behavior in the pore.

This melting process results in three distinct thermal events: (1) the eutectic melting in the pore, (2) eutectic melting in the bulk phase, and (3) ice melting in the bulk phase, which corresponds

to three peaks in the thermograms observed in the melting curves of solutions with higher concentrations (but below the eutectic molality), e.g. the 3.2 m $CaCl_2$ solution and the 3.0 m NaCl solution, respectively, shown in Figure 4 of the main article. Notably, due to the exchange of water molecular and ions between the pore and the bulk, the continuous ice melting process (following line BE) is not observed within the confined space.

For initial concentrations above the eutectic composition, the freezing process starts with the precipitation of salt resulting in a decreasing concentration an diffusion outward. However, also in this case, the concentration approaches the eutectic both in pore and in the bulk. Apart from the two eutectic signals, the third thermal signal represents then the continuous dissolution of the salt as illustrated in Figure 4 of the main article for the very concentrated solutions (4.5 m and 6 m $CaCl_2$ and 6.1 m NaCl).

S3. Model Introduction

Given the cylindrical geometry of SBA-15, the volume fraction f_V of the interfacial layer can be expressed by the following equation:

$$f_V = \frac{V_\mathrm{i}}{V_\mathrm{P}} = 1 - \left(\frac{r-t}{r}\right)^2 \tag{1}$$

where V_i is the volume of the interfacial layer, V_P is the pore volume, r is the pore radius and t is thickness of the interfacial layer, assumed to be 0.6 nm.

Assuming that all ions preferentially accumulate within the interfacial layer, the local salt molality m_i in this region can be estimated by:

$$m_\mathrm{i} = \frac{m_0}{f_V} \tag{2}$$

where m_0 is the starting molality of the salt solution, i.e., the molality averaged over the entire pore volume.

References

(1) Talreja-Muthreja, T.; Linnow, K.; Enke, D.; Steiger, M. Deliquescence of NaCl Confined in Nanoporous Silica. *Langmuir* **2022**, *38*, 10963–10974.
